# Revision Surgery Technique in the Treatment of Refractory Subcutaneous Cerebrospinal Fluid Collection Combined with Intracranial Infection Following Posterior Fossa Surgery

**DOI:** 10.7759/cureus.10610

**Published:** 2020-09-23

**Authors:** Weilong Ding, Hua Chen, Yongsheng Xiang, Jiancheng Liao, Xiaoming Qi, Xiangyu Wang, Jason H Huang

**Affiliations:** 1 Neurosurgery, The First Affiliated Hospital of Jinan University, Guangzhou, CHN; 2 Neurosurgery, Zhongshan Torch Development Zone Hospital, Zhongshan, CHN; 3 Division of Clinical Research, Baylor Scott & White Health Research Institute, Temple, USA; 4 Neurosurgery, Baylor Scott & White Medical Center, Temple, USA

**Keywords:** subcutaneous collections, cerebrospinal fluid leakage, intracranial infection, posterior fossa surgery, revision operation

## Abstract

Objective

Cerebrospinal fluid (CSF) leakage remains the most common and serious complication following posterior fossa surgery. Persistent subcutaneous CSF collections can cause wound dehiscence and predispose patients to intracranial infection. Management with conservative treatment fails in up to 40% of patients, and revision surgery remains the last resort. We hereby introduce a novel surgical technique using muscle graft or pedicled trapezius muscle flaps to repair dura and skull base defect for the treatment of subcutaneous CSF collections refractory to conservative management.

Methods

A retrospective chart review was conducted for six patients who presented to our institution from 2012 to 2020, with subcutaneous CSF collections following posterior fossa surgeries and had undergone revision surgeries after unsuccessful management with conservative treatments. Patient demographics, etiologies, culture results, revision procedures, follow-ups, and recurrences of fluid collections were collected.

Results

Of these six patients, two underwent repair of dura and skull base defect with pedicled trapezius muscle flaps, and four had arachnoid fistula repaired with autologous muscle graft. All six patients fully recovered. CSF leakage and subcutaneous fluid collections were resolved. No recurrences occurred upon the last follow-ups.

Conclusion

A revision surgery using muscle graft or pedicled trapezius muscle flaps to repair the dura and skull base defect is effective at treating persistent cerebrospinal fluid leakage and subcutaneous fluid collection refractory to conservative treatment.

## Introduction

Cerebrospinal fluid (CSF) leakage remains a serious complication that most commonly occurs following posterior fossa surgery and presents as subcutaneous CSF fluid collection (aka pseudomeningocele) [1-3]. Persistent subcutaneous CSF collection can cause wound dehiscence and leakage of CSF through the incision, which then leads to wound and/or intracranial infection [2]. Conservative treatments consist of head elevation, antibiotics, acetazolamide, and applying local pressure with compression bandage [4]. When conservative treatments fail as initial management, aspiration of the subcutaneous fluid collection, continuous lumbar drainage or lumboperitoneal CSF shunts can be employed [5]. Revision surgery remains the last resort if all other measures fail, which is also the treatment of choice for patients with intracranial infection.

Management of this difficult-to-treat complication has been documented in the literature [5-7]. However, revision surgery techniques have rarely been described, especially for cases combined with intracranial infection. We hereby describe a unique surgical technique repairing CSF leakage with pedicled muscle flap covering the extensive defect in the posterior fossa.

## Materials and methods

Institutional review board (IRB) exemption has been obtained and informed consents were waived. A retrospective chart review was conducted for six patients who underwent revision surgeries for subcutaneous CSF collections complicated with intracranial infection after posterior fossa surgeries from March 2012 to February 2020 at our institution. All patients recovered well initially but developed subsequent complications of subcutaneous CSF collections. Revision operations were performed by neurosurgery faculty members at our institution. Table 1 shows the original neurosurgical procedures for all six patients, patient demographics, etiologies, culture results, revision procedures, follow-ups, as well as recurrences of fluid collections that were captured.

**Table 1:** Summary of Six Patients with Postoperative Persistent Subcutaneous CSF Collections Complicated with Intracranial Infection

No.	Age (years)/Sex	Etiology	Initial Surgery	CSF Culture	Revision surgery	Follow-up	Collections Recurrence
1	34/m*	Vestibular schwannoma	Vestibular schwannoma resection, decompressive craniectomy	CN***	Trapezius pedicled muscle flaps	3 months	no
2	31/m	Cerebellar hemorrhage	Hematoma evacuation, decompressive craniectomy	Enteroaerogen	Muscle graft	6 months	no
3	54/m	Brainstem hemorrhage	Hematoma evacuation, decompressive craniectomy	Candida albicans	Muscle graft	14 months	no
4	53/m	Traumatic brain injury	Resection of contusion, decompressive craniectomy	Acinetobacter baumannii	Trapezius pedicled muscle flaps	7 months	no
5	56/m	Brainstem hemorrhage	Hematoma evacuation, decompressive craniectomy	Klebsiella pneumoniae	Muscle graft	1 month	no
6	44/f**	Meningioma	Meningioma resection, decompressive craniectomy	CN	Muscle graft	8 years	no

Surgical procedures

**Figure 1:**
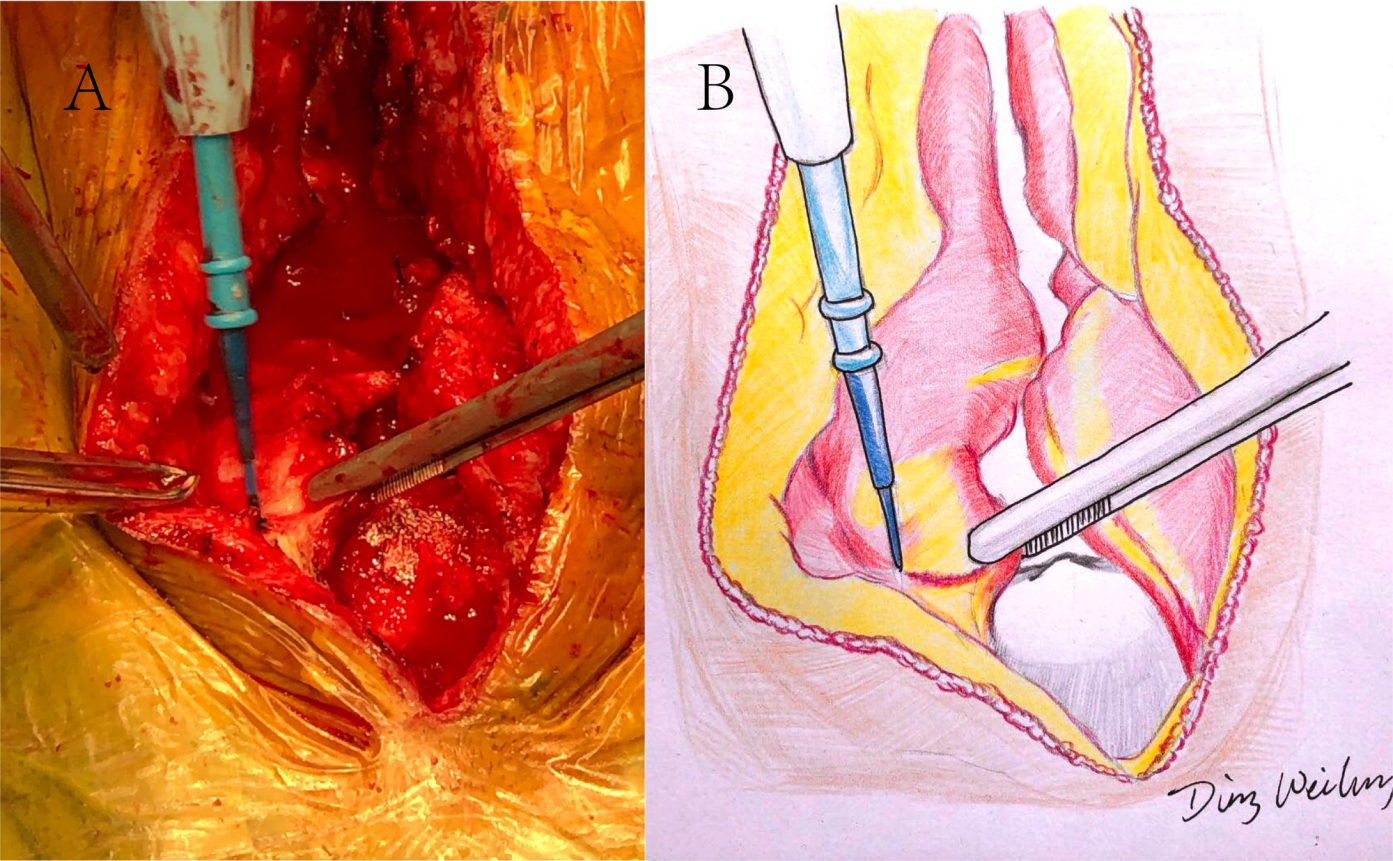
Separation of pedicled muscle flaps from the neck muscles A: Separating a layer of pedicled muscle flaps from neck muscles on both sides. B: An artist’s drawing of the original image seen in A

The patient was placed in a prone position with head secured on a surgical head clamp. An incision was made along the original incision. Infected tissue, hyperplastic connective tissue, and foreign objects including residual bone wax and silk thread were removed. Once the artificial dura mater from the original surgery was removed, the arachnoid fistula was located and filled with a small muscle graft harvested from the neck muscle (patient 2, 3, 5, and 6). The muscle graft was then carefully fixed to the thickened arachnoid with an absorbable silk suture. A layer of pedicled muscle flap (approximately 0.5 cm in thickness) was separated from the neck muscles bilaterally from top to bottom with a monopolar electrode (Figure 1). Edges of the two muscle flaps were sutured together with absorbable sutures, like closing double doors.

For patients with substantial skin and muscle atrophy (patient one and patient four), it was infeasible to suture the muscle adjacent to the incision. In this case, the dura mater defect was repaired with a pedicled trapezius muscle flap. A pre-designated area of the ipsilateral trapezius muscle was dissected and freed with fascia preserved to retain blood supply. The pedicled trapezius muscle was then flipped to ensure complete coverage of the skull defect and then fixated to the surrounding muscles with absorbable suture. A strip of Tissue Patch™ sealant film (an absorbable polymer) was applied to the suture to prevent CSF leakage. The neck muscles on both sides were then crossed tightly and closed in anatomical layers, followed by skin closure. 

During the procedure, a CSF sample was collected for Gram’s stain, culture, and sensitivity test. A course of antibiotics was administered based on the results of culture and sensitivity. The patient was instructed to maintain a lateral position to avoid compression on the incision. The continuous lumbar drainage was kept for seven to 14 days with 200mL being drained every day. After the lumbar drain was removed, a head CT or MRI was obtained. During recovery, a positive nitrogen balance diet plan was followed.

## Results

The mean age was 45.3 (31 to 56) years and intracranial infection was diagnosed in all six patients. In four patients (66.7%) intracranial infection was diagnosed based on positive CSF culture results (Table 1) while in the other two patients it was diagnosed based on clinical symptoms and laboratory testing for complete blood count, C- reactive protein, and erythrocyte sedimentation rate despite negative culture results, . The mean overall follow-up was 21.2 months (range: one to 96 months). At one month follow-up, patient four presented with hydrocephalus and required a ventriculoperitoneal shunt. Intracranial infection was eradicated in all patients based on the latest CSF laboratory testing. All subcutaneous CSF collections have been resolved according to imaging studies obtained at the last follow-up visit.

Illustrative cases

Patient One

**Figure 2:**
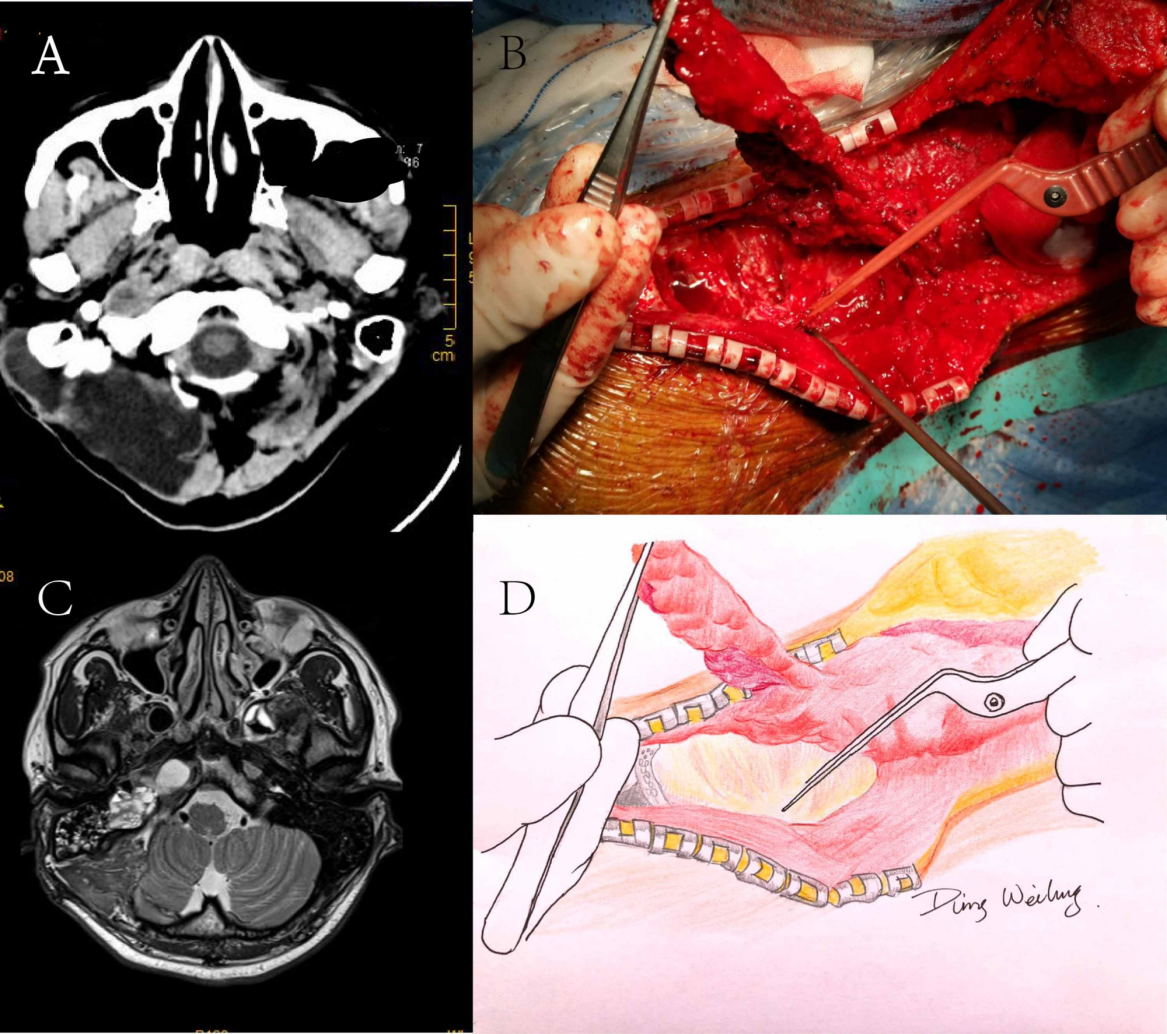
Case 1 images and illustration. A: Axial CT image showing right posterior fossa subcutaneous collection. B: Freed trapezius muscle with the fascia and flipped the pedicled trapezius muscle to cover the surface of the defective skull. C: Axial T2-weighted MRI image following one month after discharge. D: An artist’s drawing of the original image seen in B.

A 34-year-old male presenting with hearing loss was diagnosed with right giant vestibular schwannoma and underwent tumor resection via retrosigmoid transmeatal approach. Three days after discharge, he presented with fever and vomiting. Clear fluid leakage from the incision was noticed. Head CT showed right posterior fossa subcutaneous collection (Figure 2A). A continuous lumbar drain was placed and kept for a month, but the CSF leakage persisted. A revision surgery was then deemed necessary. Owing to the thin skin and muscle tissue around the incision, as well as the large skull defect, a repair with pedicled trapezius muscle flaps, was performed. An incision was made following the scar from the original surgery, and infected tissue was debrided. The pre-designated area of the trapezius muscle with the fascia was freed. The pedicled trapezius muscle was flipped to ensure complete coverage of the skull defect (Figure 2B, 2D), with part of the muscle tissues packed into the defect. The muscle flap was sutured firmly to the surrounding muscle using absorbable sutures, and a strip of Tissue Patch™ sealant film was applied. Continuous lumbar drainage was kept for two weeks. CSF culture returned negative, and recovery was uneventful. MRI at one-month follow-up revealed resolution of CSF collection (Figure 2C).

Patient Two 

**Figure 3:**
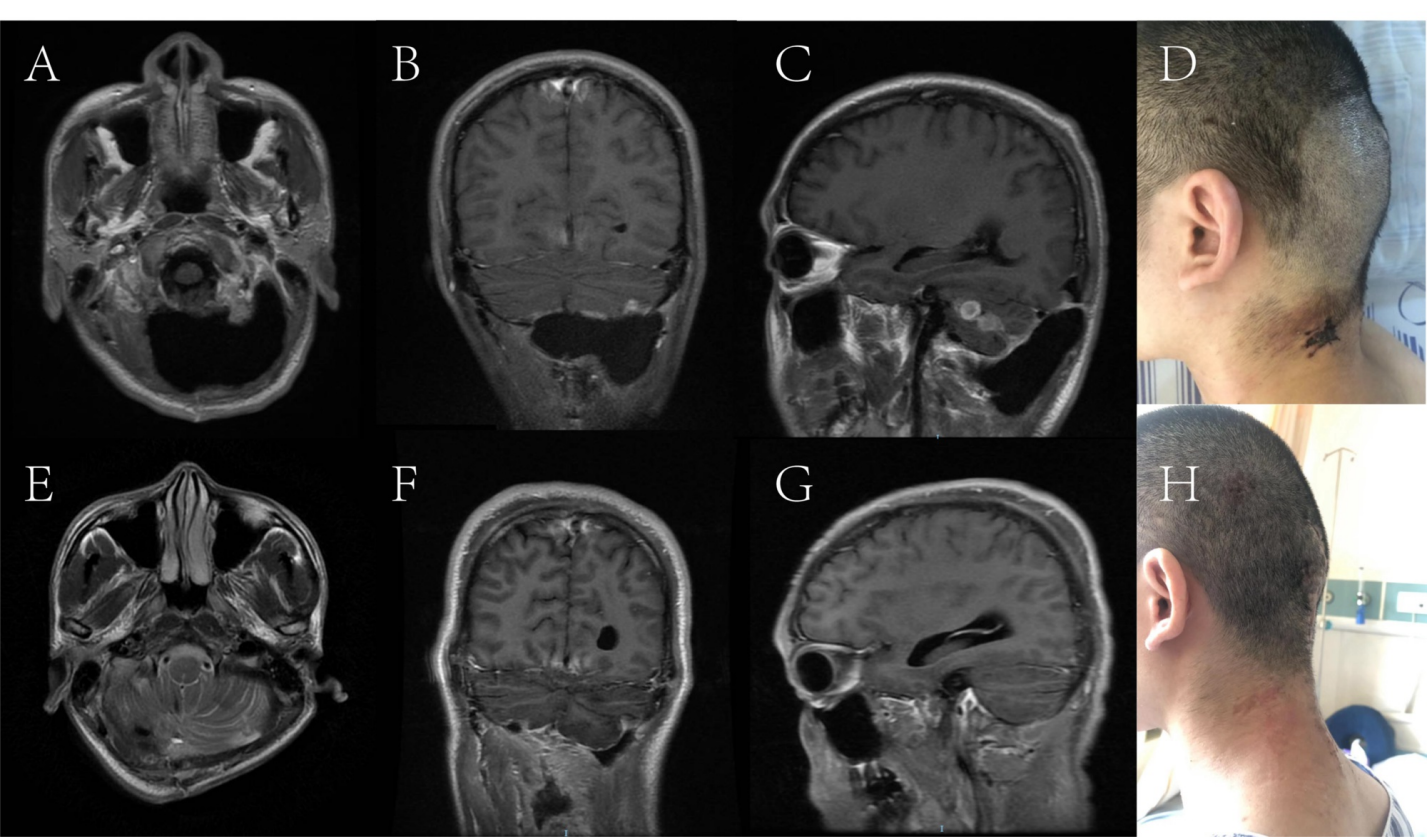
Case 2 images and illustration. A B and C: T1-weighted MRI image showing left posterior fossa subcutaneous CSF collections. D: A prominent bulge in the left occipital region. E F and G: T1-weighted MRI image following two weeks after operation showed that the subcutaneous CSF collections disappeared completely. H: The bulge in the left occipital region resolved.

A 31-year-old male presented with fever, severe headache, and vomiting with a history of posterior fossa decompression and unilateral suboccipital craniectomy (Figure 3). Physical examination revealed a prominent bulge in the left occipital region (Figure 3D). A left occipital subcutaneous fluid collection was diagnosed based on clinical findings and imaging studies. Initially, it was managed with conservative treatment including aspiration of subcutaneous collections, continuous lumbar drainage, and local compression. However, the subcutaneous fluid collections recurred once the lumbar drainage and local compression were discontinued. A revision surgery was then performed. There were two critical steps in this case. The first was locating the arachnoid fistula, which served as a valve enabling the CSF leak constantly. The fistula was packed with a small piece of muscle graft harvested from the neck muscle. The second was separating a layer of pedicled muscle flaps from the neck muscles bilaterally (Figure 1). The two muscle flap edges were stitched together like closing doors. A long strip of Tissue Patch™ sealant film was applied to the suture. The neck muscles and subcutaneous tissues on both sides were then closed tightly in anatomical layers. Continuous lumbar drainage was kept for two weeks. CSF culture returned positive for enterobacter aerogenes, and antibiotic treatment was adjusted accordingly. The patient recovered well, and the occipital bulge reflecting the subcutaneous collection disappeared (Figure 3H).

## Discussion

Subcutaneous CSF collection is an undesirable complication of neurosurgical procedures, which occurs more frequently following posterior fossa surgeries [6]. Initial treatments include repetitive aspiration of fluid collection subcutaneously, mechanical local compression, and continuous lumbar drainage [8]. When conservative treatments fail, and subcutaneous CSF collections persist, delayed wound healing and a high risk of intracranial infection ensue [3,7,9].

Cause of subcutaneous CSF collections

The risk of CSF leakage increases after craniectomies [1,10-13], which varies from 30% to 50% and reaches as high as 65% in non-watertight dural closures [1,13,14]. Decompressive craniectomy leads to large distended CSF cistern, which consistently applies high pressure to the dura mater. Eventually, the watertight dural closure is disrupted, and CSF leakage ensues. Meanwhile, patients tend to be bed-bound for a prolonged period postoperatively in a recumbent position, which renders the posterior fossa bearing the brunt of the fluid pressure imposed by the collection [1,9,13]. Tumors size greater than 3 cm is another risk factor for subcutaneous CSF collection formation in posterior fossa surgery [3]. Additionally, excessive blood in the surgical field may cause blockade of arachnoid villi leading to increased CSF pressure [3]. This further results in persistent CSF leakage and subcutaneous fluid collection. In this study, watertight dural closure, which is critical in preventing CSF leakage, was absent as we found collapsed dura with fistula during the re-exploration.

The subcutaneous fluid collection consists mainly of leaked CSF and partially of exudate. In the absence of CSF leakage, the exudate is usually diffused and absorbed. CSF has an anticoagulative effect and increases the dissolution of fiber. Therefore, when a CSF leakage is present and continuously flowing to the subcutaneous space, wound healing would be hindered. If this persists, connective tissue would eventually enclose the subcutaneous fluid forming a smooth capsule. Prolonged epidural effusion due to the continuous expansion of the fistula may cause tissue debris foreign to the central nervous system, such as bone, muscle, and subcutaneous connective tissue, to enter the subarachnoid space. This further leads to aseptic inflammation and meningeal irritation, causing symptoms like fever, headache, and nausea.

Prevention of subcutaneous fluid collection

Proper sealing of all fistulas, watertight dural closure, and eliminating dead space are important techniques for skull base and posterior fossa neurosurgical procedures. Meticulous layer-by-layer closure of posterior muscles and using autologous graft when necessary are other surgical pearls in lowering the risks of CSF leakage. Postoperative intracranial pressure can be decreased by placing the patient in a reverse Trendelenberg or Fowler’s position. In certain cases, lumbar drainage or external ventricular drainage should be continued in the immediate postoperative period [15]. As craniectomy is associated with a higher risk of CSF leakage, a craniotomy should be considered whenever possible and technically allowed [1].

Management of subcutaneous fluid collection

Conservative treatments should be implemented initially, which include head elevation, pressure dressing, and acetazolamide, followed by aspiration of the fluid collection. However, the effective rate is less than 60%7. Lumboperitoneal shunt is another alternative in managing persistent subcutaneous CSF collection [5,8,16]. This, however, comes with complications including acute subdural hematoma, pneumocephalus, neurenteric cyst, tonsillar herniation, and shunt infection [16-19]. Lumboperitoneal shunt should be avoided in patients with intracranial infection. When all other treatments fail, re-exploration is indicated.

Three critical points in this revision surgery technique are: (1) locate the fistula serving as a unidirectional valve which enables CSF leakage, and pack the fistula with a muscle graft; (2) use autologous muscle graft and muscle flap to avoid rejection; (3) maintain the tension of muscle graft and flap to eliminate dead space and further impede fluid collection formation. The choice of muscle graft or pedicled muscle flap depends on the clinical scenario. In patient one, the occipital muscle atrophy was substantial. Therefore, pedicled trapezius muscle flaps were used to cover the dura and skull defect. In patient two, there was no considerable occipital muscle atrophy. Therefore, a muscle graft was used to pack the defect.

Postoperative continuous lumbar drainage should be kept for seven to 14 days, with 200 mL CSF drained daily in all patients. This decreases intracranial pressure, prevents CSF leaking and accumulating in the subcutaneous space, and serves as an outlet of infectious CSF and a portal for intrathecal antibiotics injection.

## Conclusions

Subcutaneous CSF collection is a difficult-to-treat complication after neurosurgical procedure, as it hinders wound healing and predisposes patient to intracranial infection. When conservative management fails and/or subcutaneous CSF collection persists despite conservative efforts, re-exploration and revision surgery are mandatory. The unique surgical technique described here is effective at treating refractory subcutaneous CSF collections combined with intracranial infection following posterior fossa surgery.
